# The native reed‐specific bird, reed parrotbill, has been detected in exotic smooth cordgrass

**DOI:** 10.1002/ece3.10417

**Published:** 2023-08-09

**Authors:** Wu Dawei, Wang Zhenqi, Hu Wei, Lu Changhu, Chen Pan

**Affiliations:** ^1^ College of Biology and the Environment Nanjing Forestry University Nanjing China; ^2^ College of Life Sciences Anhui Normal University Wuhu China

**Keywords:** biological invasion, habitat use, reed parrotbill, smooth cordgrass

## Abstract

After the smooth cordgrass *Spartina alterniflora* invaded coastal China, native birds started avoiding the green desert, and bird diversity declined. After many years, a few passerine birds began to enter and use smooth cordgrass, but only birds with a nonspecialised habitat. In this study, we found that a native reed‐specific bird, the parrotbill *Calamornis heudei,* flocked and sang in a smooth cordgrass habitat throughout the overwintering period near Sheyang Port in Yancheng, Jiangsu Province. This observation suggests that native obligate birds may be forced to adapt to exotic smooth cordgrass habitats after long‐term coexistence, which would, obviously, affect the distribution, feeding and reproduction of birds. The concern is that this could be an ecological trap, leading to unknown consequences. More research is required to examine the process occurring along the Chinese coast.

## Graphical abstract

We first found that a native reed‐specific bird, the Parrotbill *Calamornis heudei*, flocked and sang in a smooth cordgrass habitat. This phenomenon indicates that native obligate birds may be forced to adapt to exotic smooth cordgrass habitats after long‐term coexistence. Obviously, the distribution, feeding and reproduction of birds would be affected, which may lead to unknown ecological consequences.

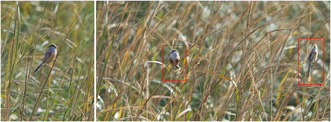

## 1 INTRODUCTION

Due to the impact of human activities, nonnative plant invasions are increasing worldwide (Dawson et al., 2017; Seebens et al., 2017; Stewart et al., 2021). This causes changes in local species biodiversity and habitats (Blakeslee et al., 2011; Doherty et al., 2016; Pejchar & Mooney, 2009; Vilà et al., 2011). The smooth cordgrass *Spartina alternifera* is a perennial graminoid C4 plant with a well‐developed root system that is native to the Atlantic coast of North America (Wang, 2006). In the 1970s, it was introduced into the east coast of China because of its good silting promotion and land building and wave elimination berm function (Zuo et al., 2012). In the 40 years since its introduction, it has expanded to more than 10 provinces and cities along the coast of China (Deng et al., 2006; Yang, 2020). Smooth cordgrass was added to the first list of 16 invasive alien species in 2003, requiring its complete removal from China's coastal areas (State Environmental Protection Administration & Chinese Academy of Sciences, 2003).

Studies have shown that smooth cordgrass invaded the coastal areas of China, occupying the bare beach and shrinking the growth space of native vegetation, resulting in the decline of original biodiversity due to changes in the habitat (Yu et al., 2022; Zuo et al., 2012). Some native passerine birds have begun to adapt to invaded habitats; they enter smooth cordgrass and rely on exotic plants for foraging, hiding, flocking, perching and reproduction (Chen et al.,  2022, 2023). This change may be due to the length of time of smooth cordgrass invasion in native habitats (Chen et al., 2019, 2022). The shrinkage of native habitats due to smooth cordgrass invasion and human disturbances may drive more birds that rely on native vegetation (Reed, *Phragmites australis*) to attempt to use the invaded habitats, although invasive vegetation may be an ecological trap (Chen et al., 2019; Ma et al., 2014). Previous studies and reports of native birds entering smooth cordgrass focused nonspecialised birds, which are widely distributed in different habitats and have strong adaptability (Chen et al., 2019, 2022, 2023; Ma et al., 2014). Obligate birds are highly dependent on specific habitats due to their narrow niche and, therefore, are more affected by exogenous disturbance (Dehling et al., 2021; Jorge et al., 2014).

The reed parrotbill, *Calamornis heudei*, belonging to the Passerine family, is a small, rare bird species inhabiting obligate reed wetlands (Lynes, 1914; Zheng, 2017). The reed parrotbill is an endemic species in China and is distributed in the reed wetlands of the eastern coast of China and the middle and lower reaches of the Yangtze River (Zheng, 2017). It is listed as Near Threatened (NT) on the IUCN Red List due to the possible decline of its suitable habitat (native contiguous reeds) (BirdLife International, 2017) and a national level II key protected animal in the newly adjusted ‘List of National Key Protected Wild Animals’. It is highly dependent on the reed habitat, and all stages of life history (including nesting, hatching, brooding and overwintering) are completed in reeds (Xiong & Lu, 2013). The bird only inhabits large reeds in freshwater rivers, lakes and coastal areas. During the breeding season, they prefer to use a mixture of new, old and dense reeds for nesting (Lynes, 1914; Xiong & Lu, 2013). In the unharvested reed habitat, the density of reed parrotbills was significantly higher than that in the harvested reed habitat (Boulord et al., 2011). The reed parrotbill is an obligate insectivorous bird. During the breeding season, they mainly feed on Orthoptera and scale insects on the surface of the reeds and in the stems (Xiong & Lu, 2014; Zheng, 2017). In winter, they mainly feed on the overwintering eggs in dry reeds (Xiong & Lu, 2013, 2014). The reed parrotbill is a monogamous bird. They usually start to nest in May, and each nest generally has five eggs (Lynes, 1914; Zheng, 2017). They breed once or twice annually (Lynes, 1914; Zheng, 2017). The male and female parents take turns incubating eggs, and the brooding period is approximately 12 days (Lynes, 1914). When they are in danger, they usually cluster escape, exhibit guardsman behaviour and issue warning calls; therefore, it is very difficult to observe their behaviour (Wang, 2018).

## 2 MATERIALS AND METHODS

**FIGURE 1:**
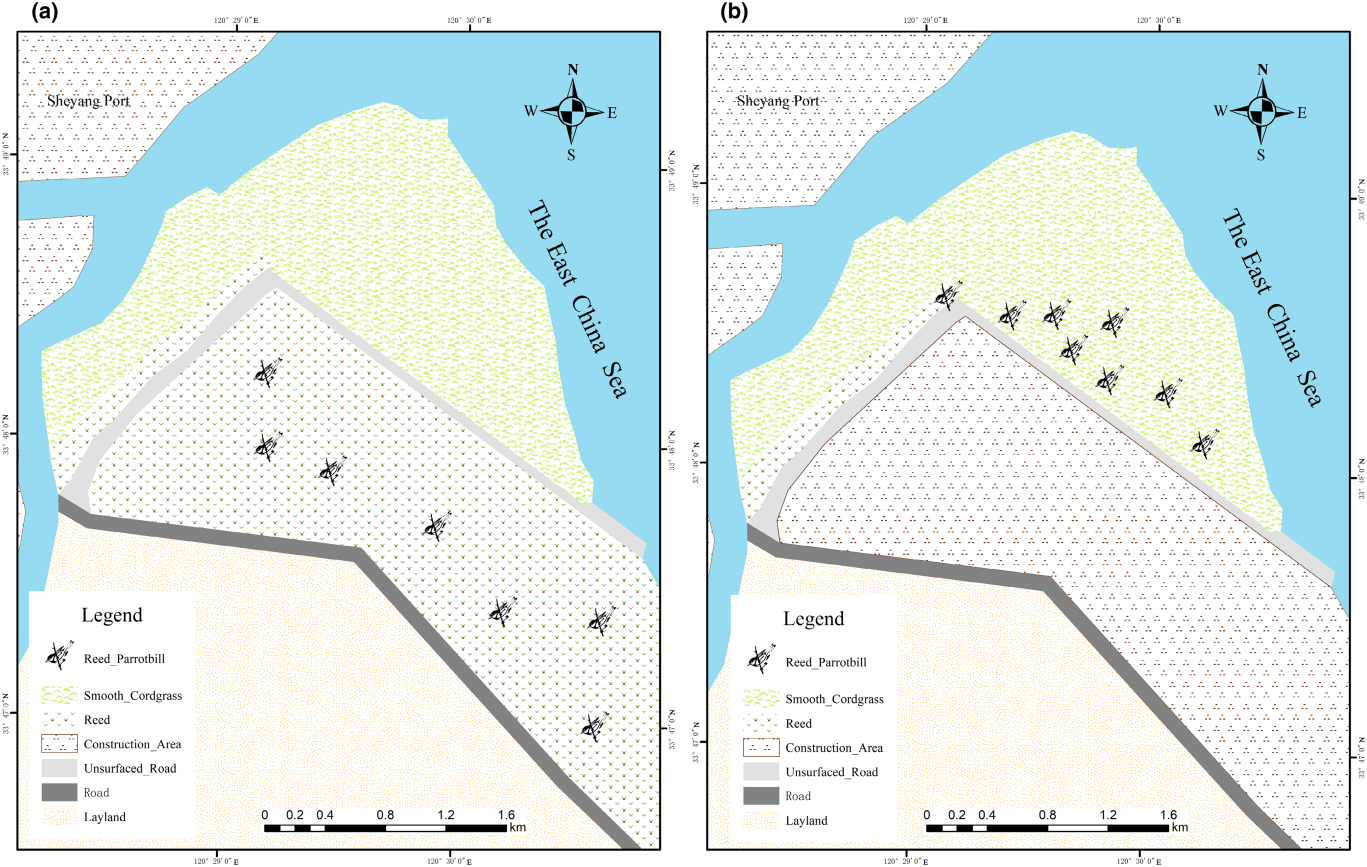
Distribution range of the reed parrotbill (a, Before the overwintering period of 2021; b, the overwintering period of 2021).

The study site is in a Spartina swamp (E 120°29′50.00″; N 33°48′35.17″) near the south of Sheyang Port in Yancheng City, Jiangsu Province. The area is approximately 3.78 km^2^, and the perimeter is approximately 11 km (see Figure 1). The first discovery of reed parrotbill entering smooth cordgrass was at noon in November 2021. We fortuitously heard the chirping of the reed parrotbill.

Over the next few months, we travelled to the area to investigate the reed parrotbill, on average once a month, and each study lasted at least 30 min. We recorded the method of detection and the number of the reed parrotbill each time. We used a transect walk to count the number and a Nikon D6 camera (600×) to record the birds. If the birds move together and flocking, we record as cluster. The bird moves alone, we record as individual.

## 3 RESULTS

**FIGURE 2:**
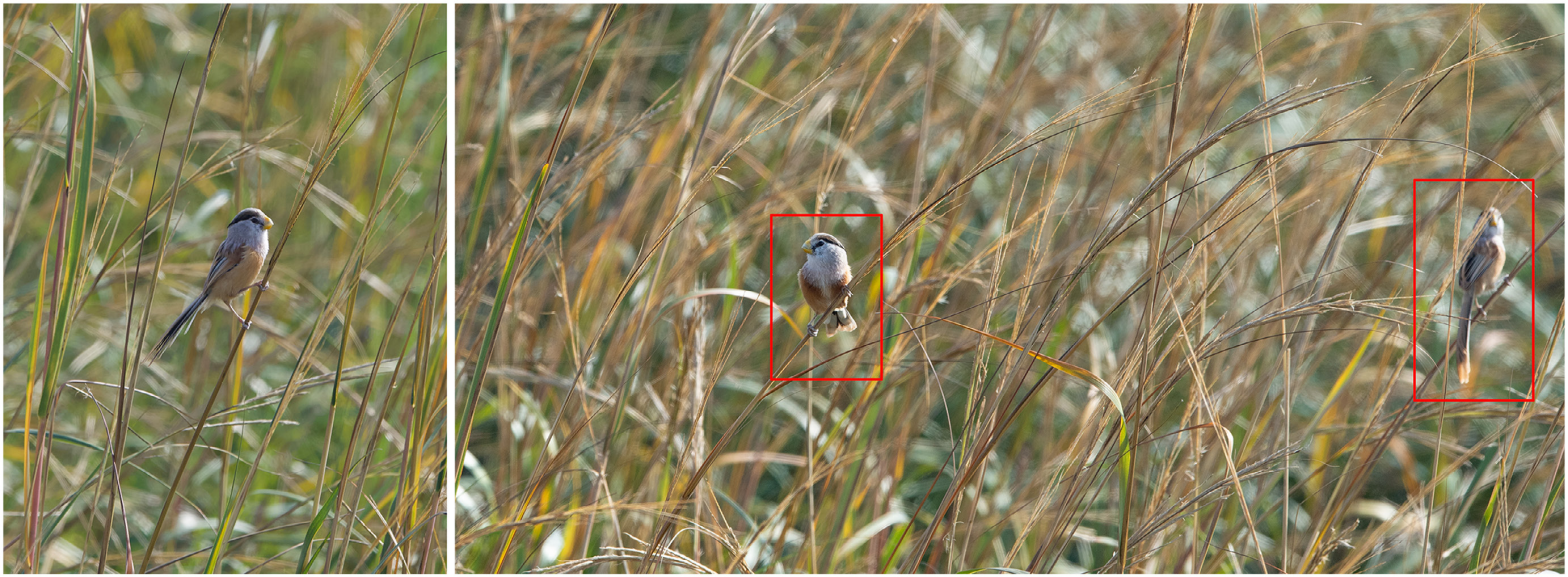
Reed parrotbill flocking in smooth cordgrass during the overwintering period.

The first discovery of reed parrotbill entering smooth cordgrass occurred at noon in November 2021, and we heard reed parrotbill chirping in smooth cordgrass. We stopped moving and stood still, then saw the reed parrotbill flocking and singing in smooth cordgrass (see Figure 2). There were many reed parrotbills jumping inside the smooth cordgrass, grasping the middle and upper parts of it and moving horizontally along the plants. From November 2021 to March 2022, throughout the overwintering period, we made five observations and found that the reed parrotbill was active in smooth cordgrass every time (see Table 1).

**TABLE 1:** Investigation time and results.

Date	Time	Method of detection	Number	Age
2021.11.12	11:08	Hearing	Cluster 12	Adult
2021.12.20	15:45	Hearing	Cluster 10	Adult
2022.01.08	07:00	Sight	Individual 6	Adult
2022.02.16	07:15	Sight	Cluster 8	Adult
2022.03.17	16:24	Hearing	Individual 4	Adult

## 4 DISCUSSION

More native birds will make use of invasive plants as the invasion period lengthens, since some native species will gradually adapt to them and use them for habitat construction, foraging and other activities (Blackburn et al., 2011; Graves, 2019; Schirmel et al., 2016). Different birds have different adaptive abilities, and a few birds with flexible behaviours and a wide range of diets can adapt and exploit new invasive habitats faster and better (Dylewski et al., 2019). After smooth cordgrass invaded from the east coast to the west coast of the United States, over time, birds, such as the clapper rail *Rallus crepitans*, Merida wren *Cistothrus meridae* and song sparrow *Melospiza melodia*, also started breeding in invasive habitats (Delach, 2006; Lampert et al., 2014; Nordby et al., 2009). In Chongming Dongtan, Shanghai, China, the nonnative marsh grassbird, *Helopsaltes pryeri*, entered and nested in smooth cordgrass (Ma et al., 2014). In Yancheng, Jiangsu, records indicate that the native plain prinia, *Prinia inornata*, adapted to and utilises smooth cordgrass, entering and singing during the breeding season (Chen et al., 2022). Less competition and dense vegetation may attract native species, but reduced food availability, tidal inundation and increased predation risk make smooth cordgrass a potential ecological trap (Chen, 2023; Nordby et al., 2009; Sun et al., 2019). Although native birds enter smooth cordgrass to reproduce, their nests are more likely to be destroyed by mammals, and their reproductive success rate is significantly reduced (Fisher & Davis, 2011; Hao et al., 2015; Nordby et al., 2009). Moreover, the courtship calls of native birds entering smooth cordgrass have changed, which greatly reduces the courtship success rate (Chen et al., 2022).

Studies and reports have shown that the reed parrotbill is a bird that is highly dependent on reed wetlands for survival, and all stages of its life cycle are completed in reeds (Xiong & Lu, 2013). It prefers the use of a mixture of old, new and dense reeds (Boulord et al., 2011). During the entire overwintering period, we found that the reed parrotbill entered smooth cordgrass, flocking and chirping. There used to be a large area of reeds in that area, and the invasive smooth cordgrass is located on the other side of the unsurfaced road near the coast. Smooth cordgrass likely invaded at least 40 years ago, and there is a mixture of dead and new smooth cordgrass. Dense vegetation can also support behaviours such as perching and singing to some extent (Chen et al., 2022, 2023; Lampert et al., 2014; Ma et al., 2014). We hypothesise that the original reed habitat was destroyed by human interference and that the reed parrotbill was forced to enter the smooth cordgrass. The reed parrotbill in this area passively entered smooth cordgrass, but eventually evolved behaviours to adapt to smooth cordgrass. Smooth cordgrass may provide an important overwintering place for the reed parrotbill, but further research on habitat quality (e.g. survival and food availability) is needed. Up to this point, we have not found any nests in smooth cordgrass, but the native vinous‐throated parrotbill does nest in smooth cordgrass (it is in the same family as the reed parrotbill, and they both rely on reeds to nest) (Chen, 2023). Therefore, further observation and research are needed.

At present, to suppress the spread of smooth cordgrass with the goal of completely removing it, the Chinese government employs mainly physical measures, such as manual uprooting, mowing, shading, flooding and burning, supplemented by chemical measures (Tang et al., 2021; Xie et al., 2019). Based on the results of an experiment in the removal project area, the species and number of birds increased significantly after smooth cordgrass was completely removed, but the change had a greater negative impact on macrobenthos (Ma et al., 2017). In addition, the use of physical methods has a few disadvantages: It requires multiple removals, the cost is relatively high, and it may lead to secondary intrusions (Zhao et al., 2022). However, simple removal of chronic invasive plants may sometimes not be an appropriate management measure. On the east coast of the United States, there is a longer history of invasive smooth cordgrass and hybridisation with native spartina; the clapper rail has become acclimated to the new spartina and has become increasingly dependent on this habitat (Lampert et al., 2014). The removal and management of smooth cordgrass has even seriously threatened the normal reproduction of this species (Lampert et al., 2014). The discovery that the reed parrotbill, a specific bird, flocks and sings in the invasive smooth cordgrass suggests that what occurred in North America may now be happening to coastal birds in China, and simple removal may not be suitable for the long‐term invasion of smooth cordgrass. More targeted and comprehensive research is required to find suitable ways to manage smooth cordgrass.

## AUTHOR CONTRIBUTIONS

**Wu Dawei:** Data curation (lead); formal analysis (lead); investigation (equal); software (lead); writing – original draft (lead); writing – review and editing (equal). **Wang Zhenqi:** Investigation (equal); resources (lead); visualization (equal); writing – review and editing (equal). **Hu Wei:** Data curation (equal); investigation (equal). **Lu Changhu:** Conceptualization (lead); funding acquisition (lead); project administration (lead); supervision (lead); validation (lead); writing – review and editing (lead). **Chen Pan:** Conceptualization (lead); data curation (equal); investigation (equal); methodology (equal); validation (lead); writing – review and editing (lead).

## CONFLICT OF INTEREST STATEMENT

The authors have no conflicts of interest to declare.

## Data Availability

Data sharing is not applicable to this article because no new data were created or analysed in this study.
